# The Tailored Implementation in Chronic Diseases (TICD) project: introduction and main findings

**DOI:** 10.1186/s13012-016-0536-x

**Published:** 2017-01-10

**Authors:** Michel Wensing

**Affiliations:** 1 Heidelberg University Hospital, Department of General Practice and Health Services Research, Heidelberg, Germany; 2 Radboud University Medical Centre, Radboud Institute of Health Sciences, Nijmegen, Netherlands

**Keywords:** Barriers and facilitators for implementation, Intervention development, Implementation science, Evidence-based healthcare

## Abstract

**Background:**

The Tailored Implementation in Chronic Diseases (TICD) project aims to contribute knowledge on how to improve healthcare for patients with chronic diseases and, at the same time, knowledge on concepts and methods of tailoring interventions to local conditions. In this contribution, the project is briefly introduced and its main findings are discussed.

**Discussion:**

The tailored implementation programs in the TICD project had little impact, for which we provide a range of potential explanations. Structured group interviews with informed stakeholders, such as clinicians and researchers, were used to generate perceived determinants of practice and suggestions for tailored implementation strategies. They were productive and valid, yet incomplete, if compared to perceptions of healthcare providers who received the tailored implementation programs. Ongoing monitoring of determinants of practice during intervention delivery seems required to adapt the interventions to emerging needs and opportunities.

## Background

The implementation of innovations into healthcare practice remains difficult, despite several decades of scientific research and development. It is widely felt that implementation strategies need to be tailored to local needs and opportunities for change [1]. Tailoring means “making fit with individual customers”, who are typically healthcare providers in this context. Analysis of published tailored implementation programs showed that a wide variety of approaches and methods for tailoring were applied [2], while there is little guidance on their usefulness. The Tailored Implementation in Chronic Diseases (TICD) project was a European research project, funded under the European Community Seventh Framework during the years 2011–2015. Its aim was to assess tailored programs to improve healthcare for patients with chronic diseases (mainly in primary care settings), while at the same time contributing knowledge on concepts and methods of tailoring interventions.

A study protocol describes the outline of the project in detail [3]; its mean features are summarised here. The TICD project was informed by reviews of published research on tailored implementation and an update of the Cochrane review on tailored interventions (now covering 32 studies) that was done as part of the project [4]. The empirical research in the TICD project comprised of five separate research projects, concerning multi-morbidity (Germany), vascular conditions (Netherlands), depression in the elderly (Norway), chronic obstructive pulmonary disease (Poland) and obesity (United Kingdom). In each of the countries, specific goals for improving healthcare for the targeted condition were chosen as targets throughout the project. Given the targeted chronic conditions, all studies focused on primary medical care. In each of the five countries, three studies were performed consecutively:

- an exploration of determinants of practice regarding the targeted conditions, using interviews and surveys with healthcare providers;
- group interviews with various stakeholders to collect suggestions for educational, organisational and other interventions to address determinants of practice;
- cluster randomised trials of a tailored implementation program, based on insight into determinants and interventions, and related process evaluations (evaluations of processes leading to outcomes).

The trials were statistically powered to detect effects on primary outcomes and not necessarily for post hoc analyses. The sample sizes were reasonably large (several hundreds of patients in each trial), but in line with the confirmatory approach, we have been careful with post hoc analyses of outcomes. Changes on determinants of practice, which are basically mediating or moderating factors for intervention effects, were subject of the process evaluations. “Determinants of practice” was chosen as the overall concept, which covers barriers, obstacles, facilitators and enablers for implementation. Examples include lack of knowledge, disagreement with recommendations or anticipated resistance in patients. Specific findings are presented in separate publications, most of which have been brought together in a collection of articles in *Implementation Science*. In this contribution, the main findings and lessons from the research project are discussed.

### Developing tailored interventions

Group methods, such as brainstorming and focus groups, with healthcare providers and others, were productive in terms of generating items. Many of the suggested determinants and strategies were recognised as valid by health professionals, who had been exposed to the implementation programs that were based on the group interviews [5]. This finding suggests that structured group methods in stakeholders can effectively identify determinants of practice and related interventions. Nevertheless, new items emerged during the exposure to implementation programs. Continued monitoring and adaptation of interventions during their delivery seems also required to address missed or non-prioritised key determinants, which was observed in the process evaluation. It should be noted that we measured perceptions with interviews and questionnaires in both studies; these may not completely reflect real determinants of practice and change. In pragmatic field studies in healthcare; however, it is difficult to assess determinants of behaviours feasibly in other ways.

We had not anticipated the large volume of items generated in the interviews, so we had not planned for this and used a pragmatic method for choosing key determinants and strategies for addressing those. Items were clustered in conceptual categories [6], but the choice of priorities focused on single items. More systematic methods for prioritisation of items are recommended to future projects. On the other hand, different stakeholders seemed to provide largely the same ideas in interviews, although an important caveat is that we did not involve patients in all countries. The involvement of various stakeholders (clinicians, patients, purchasers, researchers) may have benefits for the perceived credibility of the chosen interventions, but this has to be balanced with the additional effort and costs. It may be most crucial for the credibility of a program to involve the targeted groups in the final choice of interventions. Also, some group activities may be primarily designed to enhance engagement of stakeholders rather than to add information that is crucial for the design of programs.

An alternative approach to tailored implementation is the use of existing theory on change of behaviour and organisations. In the TICD project, we used a broad theories-orientated framework to guide interviews and analysis [7]. Given its comprehensiveness and high level of specification, this conceptual framework is itself an important contribution to implementation science. Other researchers used more narrowly defined theories, for instance from psychology, more closely in the design and evaluation of interventions. In the TICD project, however, we felt that no theory convincingly could explain the organisation and delivery of healthcare for patients with chronic diseases. This reflects the current state of science, which is that knowledge of determinants of individual and organisational behaviours is limited.

### Tailored interventions in practice

The tailored implementation programs resulted in improvements on some outcomes, but they had overall little observable impact on primary or secondary outcomes. Table 1 summarises potential reasons for lack of effects, based on discussions in the TICD project group in February 2015. We considered these reasons plausible hypotheses.

**Table 1:** Potential reasons for lack of effects in the TICD trials

• Research evidence for some recommended clinical activities, which were targeted in the trails, is limited or mixed. Therefore, these recommendations might not have been credible for the targeted healthcare providers.
• The list of identified and targeted determinants of practice was not complete, so we might have failed to address key factors in the implementation programs. In addition, some determinants could not be adapted in the context of the TICD project, such as payments and organisation of healthcare delivery.
• The chosen implementation strategies were not sufficiently matched with targeted determinants, or not effective in the targeted groups and settings. Insight into the linkages of interventions and determinants is very limited.
• Health professionals’ agreement to participate in the implementation program was not a good predictor of intention to change behaviour. For instance, there are many competing priorities or participants may felt little ownership of the program, despite the tailoring.
• The provided tailored interventions were not used, thus could not have impact. The fidelity of the implementation programs was overall limited.
• Determinants, interventions and contextual factors interacted in complex ways, which reduced their impact. For instance, treatment targets for vascular risk may be used flexible in patients with complex morbidities. • The primary outcomes were not adequately chosen, for instance because they were largely dependent on patents’ biology, or the available measures lacked responsiveness to change.
• The follow-up period in the TICD trials was too short to detect change, as most changes require much more time to happen.
• The pragmatic trials involved heterogeneous populations and low control of intervention delivery, which has reduced impact and hidden impact in subpopulations.
• Contextual factors led to improvements in the control groups, thus reducing the added value of the tailored implementation programs.

It has been argued that medical care is often complex, meaning that many processes influence the outcomes in unpredictable ways. Measures can only partly capture these complexities, and measures based on clinical or administrative records may unable to catch specific changes in clinical decision making and counselling of patients [8]. The significant effects on some outcomes may be interpreted as indication that change occurred, but it should be noted that we also found evidence of deterioration on other outcomes. It may also be possible that general trends in the health systems, particularly those concerning reimbursement or politics on health professions, had impacts that overruled our implementation programs. For instance, GP-centred care programs in Germany and chronic care groups in the Netherlands are major contextual influences, also because they include additional reimbursement for some of the activities that were targeted.

It seems that the targeted health professionals used the implementation programs as a menu of options, from which they chose as felt necessary. It seems important to invest more in the uptake of the implementation interventions, perhaps by using hierarchical power or by providing strong financial incentives and thus increase implementation fidelity. However, this raises issues of professional autonomy and person-centred care. An alternative is to tailor interventions more intensively to individual users and specific organisations (“personalized implementation”), but this can be very costly. To be a realistic option for application outside scientific studies, it would require more efficient methods than individual or group interviews.

## Conclusions

The TICD project focused on improving primary medical care for patients with chronic diseases. To what extent are its findings and tools valid in other populations and settings is an open question. For instance, in some other settings, the role of managers may be crucial [9], while it is limited in primary care that is delivered in office-based practices. It seems plausible that these are generalizable across a range of healthcare settings, and possibly beyond, but this claim has yet to be supported by empirical research. Overall, the TICD project raises concerns about tailoring as a recommended approach to implementation of innovations in healthcare practice.

The project is one of the first studies on methods for tailoring strategies for improving healthcare in implementation science. The project opens a field of research, with many unanswered questions. We did not focus on the use of resources by different methods, but this is obviously an important topic for future research. The efficiency of tailored implementation may be enhanced by separating out the identification of items (which may be best done with informed key people) and other activities to involve stakeholders for enhancing the credibility of the approach. An important question is also how to prioritise determinants of practice and tailored interventions from a large number of suggested ones. It is also important to explore how tailoring (or adaptation) of interventions during their delivery is done, if at all.

It is difficult to provide firm take-home message for users of tailored interventions, given the limited evidence on tailoring methods and the absence of impact on outcomes in the trials. Some findings were striking. Structured group interviews of 2 h each with 3–7 well-informed individuals, such as healthcare providers and researchers in the field, were productive and seemed valid. Nevertheless, it would be desirable to experiment with different sampling and interview methods in future research in order to examine what is most effective and efficient. Ongoing monitoring of determinants of practice during intervention delivery seems required to adapt the interventions to emerging needs and opportunities. This is consistent with recent theoretical work, which suggests that adaptation of intervention is crucial given contextual and political changes [10]. However, it poses a tension with the requirements for summative evaluation, e.g. randomised trials, which require a high degree of standardisation of the intervention for a meaningful interpretation. Tailoring interventions to local conditions makes sense intuitively, but there is as yet no evidence to expect strong effects on outcomes.
